# Blocking ATM Attenuates SKOV3 Cell Proliferation and Migration by Disturbing *OGT/OGA* Expression *via hsa-miR-542-5p*

**DOI:** 10.3389/fonc.2022.839508

**Published:** 2022-06-20

**Authors:** Ning Wang, Miaomiao Yu, Yan Fu, Zhanchuan Ma

**Affiliations:** ^1^ Central Laboratory, The First Hospital of Jilin University, Changchun, China; ^2^ Department of Gynaecology II, The First Hospital of Jilin University, Changchun, China; ^3^ Bethune Institute of Epigenetic Medicine, The First Hospital, Jilin University, Changchun, China; ^4^ Department of Gynaecology I, The First Hospital of Jilin University, Changchun, China

**Keywords:** ovarian cancer, O-GlcNAcylation, O-GlcNAc transferase, O-GlcNAcase, miR-542-5p, ATM inhibitor

## Abstract

Blocking ataxia telangiectasia mutated (ATM), a crucial player in DNA repair responses, has been proposed as a promising strategy in anti-cancer therapy. Most previous studies have focused on DNA damage response-related pathways after administration of ATM inhibitors. However, ATM inhibition could potentially influence a wide range of changes in gene expression, which remain poorly defined. Here, we report that administration of the ATM inhibitor KU60019 led to impaired migration and enhanced apoptosis in the ovarian cancer cell line SKOV3, accompanied by abnormally elevated O-GlcNAc transferase and O-GlcNAcase expression levels. In addition, KU60019 treatment significantly suppressed expression of *hsa-miR-542-5p* in SKOV3 cells. Up-regulation of *hsa-miR-542-5p* expression inhibited increases in OGT and OGA level, and reversed the effects of ATM inhibition on apoptosis and migration in SKOV3 cells. Finally, we found aberrant expression of *OGT* and *OGA* to be associated with ovarian cancer patient survival. Taken together, our results suggest that ATM inhibition may promote SKOV3 cell apoptosis *via* suppressing *hsa-miR-542-5p* and elevating *OGT* and *OGA* expression, providing new insights into the application of ATM inhibitors in cancer immunotherapy.

## Introduction

Ovarian cancer accounts for more than 100,000 deaths worldwide, with an estimated 200,000 new cases diagnosed annually, making it one of the most common cancers among women (1). Over the past few decades, the incidence of ovarian cancer has been increasing, constituting an enormous global health burden (2). Advanced-stage ovarian cancer is correlated with a high risk of mortality, with five-year survival rates remaining below 45% (3). Although surgery, chemotherapy, and DNA repair pathway-targeting have been demonstrated as promising strategies for ovarian cancer treatment (4), this cancer’s complex molecular and genetic changes in both development and progression need to be addressed to improve treatment efficacy.

The DNA damage repair system is a well-organized molecular machinery comprising hundreds of molecules that function in preventing the occurrence of mutation in a cell (5). Therefore, errors in DNA damage repair may lead to tumorigenesis or even cell death (5, 6). Ataxia telangiectasia mutated (ATM) is one the core molecules involved in the DNA damage response, and somatic mutations in ATM account for approximately 30% of the risk of developing different types of cancers, including ovarian cancer (7). Thus, targeting ATM is considered to be an appropriate strategy for synthetic cancer cell-killing therapies, and specific ATM inhibitors such as KU60019 have been reported to be improved glioma treatment (8). In addition, a combination of KU60019 and enzalutamide re-sensitized castration-resistant prostate cancer cells to enzalutamide *via* targeting miR-421 (9). Although the functions of ATM signaling in cancer cells have been widely studied, the impact of ATM and its inhibition on other aspects of cancer cells remain unclear.

O-linked β-N-acetylglucosaminylation (O-GlcNAcylation) is an essential biological process that is involved in the modulation of many cellular cascades, including the metastasis and progression of cancers such as ovarian cancer (10), colorectal cancer (11), gastric cancer (12), and others (13). In general, O-GlcNAcylation activation in cancer cells can be catalyzed by O-GlcNAc transferase (*OGT*), whereas O-GlcNAcylation is suppressed by O-GlcNAcase (*OGA*) (14, 15). Aberrant expression of *OGT* and *OGA* have been detected in many cancers. For example, increased expression of *OGT* was found to be related to the histological grade of the tumor in breast cancer (14), and blocking O-GlcNAcylation resulted in reduced vascular endothelial factor expression and impaired angiogenesis in prostate cancer (16). These studies indicate that a central process of nutritional homeostasis might control the key signaling and metabolic pathways that regulate cancer initiation, metabolism, and progression (13).

MicroRNAs are small, noncoding, single-stranded RNAs that post-transcriptionally regulate gene expression to exert numerous biological effects. The microRNA *hsa-miR-542-5p* (*miR-542-5p*) has been linked to cell proliferation, differentiation, and apoptosis (17). It has been shown to suppress the phosphorylation of FAK/PI3K/AKT, by attenuating the proliferation and migration ability of mouse NIH-3T3 cells through directly targeting integrin α6 (18). Moreover, *miR-542-5p* expression was found to be downregulated in non-small cell lung cancer tissues, related to advanced TNM stage, vascular invasion, and lymphatic metastasis (19). We previously demonstrated that *miR-542-5p* plays a key role in breast cancer progression (20). These lines of evidence suggest that *miR-542-5p* might be a new therapeutic target for the diagnosis and treatment of cancer.

In this study, we investigated the role of *miR-542-5p* in the presence of an ATM inhibitor (KU60019) in SKOV3 ovarian cancer cells, which are widely used to investigate DNA damage response pathway-related genes. Given the accumulating evidences that tumorigenesis and cancer progression may be driven by perturbations in metabolic processes (20, 21), we further detected the effect of aberrant *OGA* and *OGT* expression, important glycometabolism-related genes, after KU60019 treatment on the malignant and metastatic behaviors (proliferation and migration) of SKOV3 cells. These findings can provide insight for improving the therapeutic effects of ATM inhibition with consideration of O-GlcNAcylation and the role of *miR-542-5p* in cancer progression.

## Materials and Methods

### Cell Culture and Drug Treatment

The ovarian cancer cell line SKOV3 was obtained from ATCC and cultured in Dulbecco’s Modified Eagle Medium (DMEM) (Sigma) supplemented with 10% v/v fetal bovine serum (FBS, Sigma), 100 U/mL penicillin and streptomycin [Yeasen, ShangHai, CN), and 2 mmol/l l-glutamine (Yeasen, ShangHai, CN)]. Cells were tested by PCR and found to be mycoplasma negative; and was authenticated by profiling of STRs (Short Tandem Repeats) analysis.

ATM inhibitor KU60019, OGT inhibitor OSMI-1 and OGA inhibitor MK-8719 were purchased from tsbiochem (tsbiochem, ShangHai, CN), and stored at -80°C in stock solution (KU60019 and MK-8719,10 mM; OSMI-1, 20mM, dissolved with dimethylsulfoxide). Then, 5 × 10^5^ cells/ml SKOV3 cells were collected during their logarithmic phases of growth and treated with 10 μM KU60019 (21) or MK-8719 (22), or 20 μM OSMI-1 (23) for 24 h.

### Transfection Assay

SKOV3 cells were plated into a 24-well cell culture plate (1×10^5^ cells/well) for 12 h. Transfection with miRNAs was completed according to the manufacturer’s (genepharma, CN) instructions. After 48 h, cells were collected and the transfection efficiency was detected by RT-PCR.

### Migration Assay

SKOV3 cell with high confluence (approximately 95%) were scratched with 200 ul pipette tips. After washing with phosphate buffer solution, the widths of cells wounds were photographed at 0 h and 24 h. Image J software was used to calculate the ratio of cell migration.

### CCK‐8 Assay

SKOV3 cells were placed at 2 x 10^3^ cells/ml into the 96‐well plate overnight. After miRNA transfection and KU60019, or OSMI-1, or MK-8719 treatment, cell proliferation was assessed using CCK‐8 solution (Yeasen, ShangHai, CN) kit according to the manufacturer’s instructions. Absorbance at 450 nM was recorded by a microplate reader.

### Dual-Luciferase Assays

A dual-luciferase assay was performed to verify the relationship between hsa-miRNA-542-5p and OGT/OGA. Luciferase vectors contain the wild or mutant fragment sequence of OGT/OGA (Genepharma, China) were transfected into HEK 293FT cells with hsa-miRNA-542-5p mimics or negative control (NC) (Genepharma, China) according to the manufacturer’s instructions of jetPRIME (Polyplus Transfection, France). After 48 hours, cell lysates were collected and Renilla luciferase activity was measured by a standard multimode plate reader (Biotek Epoch, USA), and normalized to the activity of Rluc.

### mRNA and miRNA Detection

Total RNA was extracted from the above cells with Trizol (Invitrogen) reagent. TransScript First-Strand cDNA Synthesis SuperMix (TransGen Biotech) was used to synthesize cDNA. qRT-PCR was performed to assess the relative expression of the target genes with a SYBR Green Kit (TransGen Biotech) under an ABI StepOnePlus system (Applied Biosystems). Relative mRNA/miRNA expression was calculated using the 2^−ΔΔCT^ method. The related primer sequence set is listed as follows:

**Table:** 

Primer name	Primer sequence
*h/m-actin*	F: 5’ TTCAACACCCCAGCCATG 3’ R: 5’ CCTCGTAGATGGGCACAGT 3’
*h-OGT*	F: 5’ TCCTGATTTGTACTGTGTTCGC 3’ R: 5’ AAGCTACTGCAAAGTTCGGTT 3’
*h-OGA*	F:5’ CATAGGATGTTTTGGCGAGAGAT 3’ R:5’ GGTGAGATCGCATAGATGAACTC 3’
*h-miR-542-5p*	F:5’ CTCCTCTCGGGGATCATCAT 3’ R:5’ TATGGTTGTTCACGACTCCTTCAC 3’

### Flow Cytometry Analysis

To detect the ration of cell apoptosis, SKOV3 cells in 6-well plates were treated with KU60019, or OSMI-1, or MK-8719 for 24 h. Cells were collected and stained with Annexin V-fluorescein isothiocyanate and propidium iodide according to the manufacturer’s (Yeasen, ShangHai, CN) instructions. To detect OGT/OGA expression at protein level, SKOV3 cells were collected and permeabilized with Fixation/Permeabilization Kit (eBioscience), then, the primary antibodies Anti-MGEA5/OGA antibody (Rabbit, 1:50, ab124807, Abcam) and Anti-OGT antibody (Rabbit, 1:100, ab177941, Abcam) were added to 5 × 10^5^ SKOV3 cells for 1 hour at 4°C. And cells were collected and washed with cold phosphate buffer, then, secondary antibodies conjugated with FITC (Goat, 1:2000, ab150077, Abcam) for 30 min at 4°C. And cells were collected and washed with cold phosphate buffer for OGT/OGA detection. Apoptosis ratio and OGT/OGA were detected by an Ariall flow cytometer (BD Biosciences) and analyzed by FlowJo software (Version 10; FlowJo).

### Bioinformatic Analysis

Kaplan-Meier plotter (http://kmplot.com/analysis/) was used to evaluate the prognostic value of target genes in ovarian cancer patients (24). The correlations between the target genes and overall survival (OS), first progression (FP), and post progression survival (PPS) were analyzed in ovarian cancer patients, using the hazard ratio (HR) with 95% confidence intervals (CIs) and log rank *p* value.

Target sequences in FASTA format were obtained from the U.S. National Library of Medicine’s National Center for Biotechnology Information (https://www.ncbi.nlm.nih.gov/). Then, RNA22 (https://cm.jefferson.edu/rna22/Interactive/) was used to identifying potential binding sites of *OGT* and *OGA* to *miR-542-5p* (25).

GO annotations and KEGG pathway analyses were performed using a KOBAS online analysis tool (http://kobas.cbi.pku.edu.cn/kobas3) on *OGT/OGA*, and top 10 enriched signal pathways with *p* < 0.05 were obtained (26).

Copy-number alterations analysis was performed using cBioPortal for Cancer Genomics database (http://www.cbioportal.org/).

Pathological Stage Plot was drewn by using Gene Expression Profiling Interactive Analysis (http://gepia.cancer-pku.cn/) to analyze impact of target genes in ovarian cancer development.

### Statistical Analysis

Statistical significance was determined by the unpaired, two-tailed Student’s t test analysis using Prism 7.0 (GraphPad Software). And *p* < 0.05 being considered statistically significant. Unless otherwise stated, data are calculated based on three independent experiments. Data were expressed as mean ± SD.

## Results

### KU60019 Impaired the Migration and Viability of SKOV3 Cells

**Figure 1:**
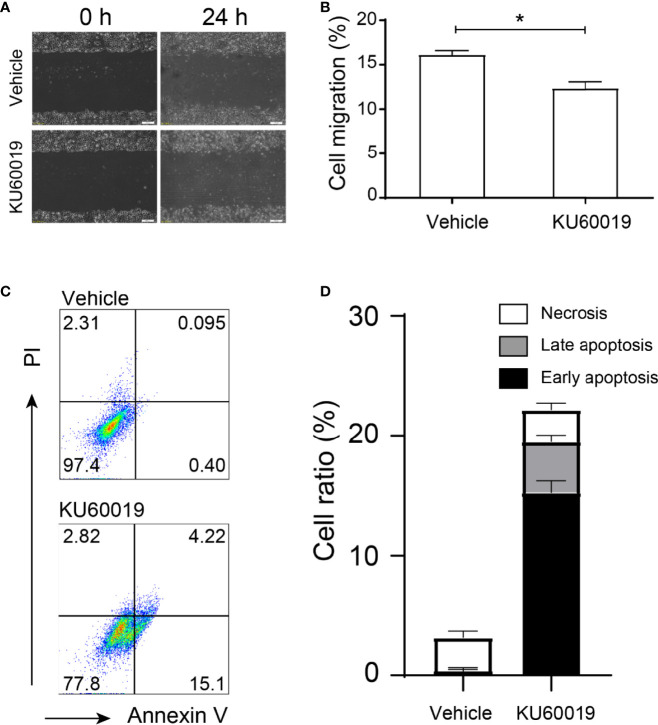
ATM blocking inhibited performances in SKOV3 cells. SKOV3 cells were treated with vehicle or 10 μM KU60019 for 24 h prior to wounding or apoptosis detection. Images were taken of each well at indicating time-points. The width of the wound was measured three times per image **(A, B)**. Cell apoptosis was detected by flow cytometry **(C-D)**. *P < 0.05. Data are representative of three independent experiments with similar results. Quantification of signal was shown in bar graphs and error bars represent mean ± SD.

SKOV3 cells were treated with vehicle or 10 μM KU60019 for 24 hours, we found that the cell migration was significantly inhibited (**Figures 1A,B**). In addition, inhibition of ATM also increased the cell apoptosis ratio *in vitro* (**Figure 1C**). Both the early and late apoptosis ratios dramatically increased in the KU60019-treated group when compared with those in the control group, which showed significant differences (**Figure 1D**). These results indicated a protective role of ATM on SKOV3 migration and proliferation.

### *Hsa-miR-542-5p* May Regulate *OGT* and *OGA* Expression in ATM-Inhibited SKOV3 Cells

**Figure 2:**
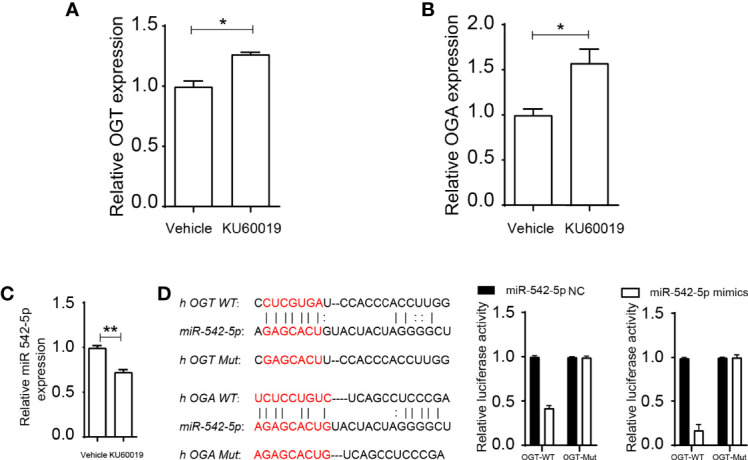
miR-542-5p could bind to OGT and OGA. SKOV3 cells were treated with vehicle or 10 μM KU60019 for 24 h. Then cells were collected, and qPCR analysis of OGT **(A)**, OGA **(B)**, and miR-542-5p(C)in SKOV3 cells. **(D)** Potential binding sites of OGT and OGA to miR-542-5p, and putative miR-542-5p.target sequence in wild-type (WT) and mutated (Mut) 3’UTR of OGT or OGA. And luciferase reporter assay was performed to confirmed the relationship between OGT/OGA and miR-542-5p. *P<0.05; **P< 0.01. Data are representative of three independent experiments with similar results. Quantification of signal was shown in bar graphs and error bars represent mean ± SD.

Inhibition of ATM upregulated the expression of *OGT* and *OGA* in SKOV3 cells (**Figures 2A, B**), an approximate 20% and 60% increase difference could be detected in OGT and OGA expression, respectively, indicating KU60019’s regulatory effect on glycometabolism. In addition, KU60019 treatment significantly restrained *miR-542-5p* expression in SKOV3 cells (**Figure 2C**). We further found that *miR-542-5p* could potentially bind to both *OGT* and *OGA* (**Figure 2D**), and the subsequent luciferase assay showed that the luciferase activity was significantly decreased after *miR-542-5p* mimics were transfected into HEK 293FT cells, verified the interaction between *miR-542-5p* and *OGT/OGA*. This may lead to changes in cell performance. Indeed, we found that *OGT* and *OGA* were mainly enriched in glycoprotein metabolic processes (**Tables 1**, **2**), suggesting central roles of *OGT* and *OGA* in regulating cellular glycometabolism cascades. Collectively, these results suggested that *miR-542-5p* might target *OGT* and *OGA* to regulate the expression profile in SKOV3 cells during KU60019 treatment.

**Table 1:** GO analysis of OGT/OGA associated genes with ovarian cancer.

Term	Description	Count	*p*-value	adj p-values
GO:0016032	viral process	456	0.000136	0.00244
GO:0061087	positive regulation of histone H3-K27 methylation	5	0.000306	0.00244
GO:0004415	hyalurononglucosaminidase activity	7	0.000408	0.00244
GO:0006517	protein deglycosylation	8	0.000459	0.00244
GO:0043995	histone acetyltransferase activity (H4-K5 specific)	9	0.00051	0.00244
GO:0035020	regulation of Rac protein signal transduction	10	0.00056	0.00244
GO:0009100	glycoprotein metabolic process	11	0.000611	0.00244
GO:0046626	regulation of insulin receptor signaling pathway	13	0.000713	0.00244
GO:0006111	regulation of gluconeogenesis	14	0.000764	0.00244
GO:0043982	histone H4-K8 acetylation	16	0.000866	0.00244

**Table 2:** KEGG pathway analysis of OGT/OGA related pathways with ovarian cancer.

Term	Description	Count	*p*-value	adj p-values
hsa04931	Insulin resistance	108	0.00000778	0.0000701
hsa00514	Other types of O-glycan biosynthesis	22	0.00117	0.00527

### Up-Regulation of *miR-542-5p* Reversed the Overexpression of *OGT* and *OGA* in SKOV3 Cells When Blocking ATM

**Figure 3:**
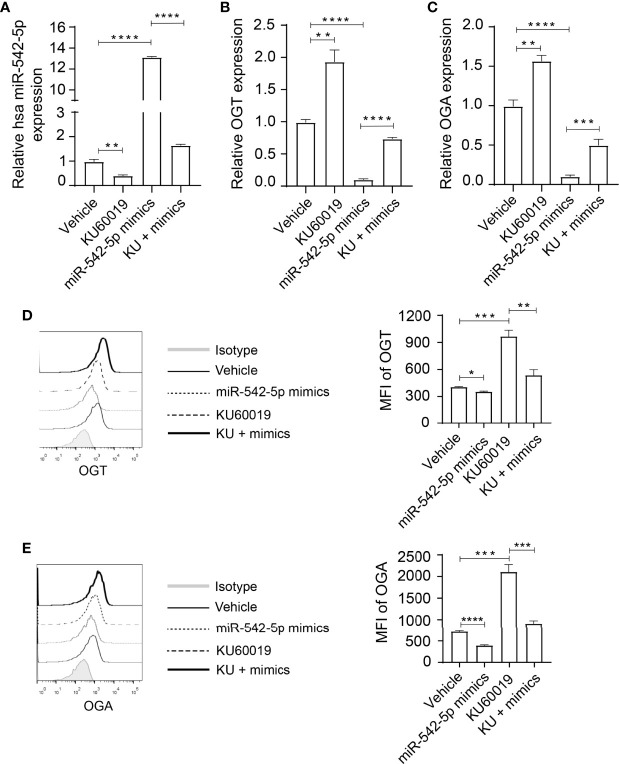
Elevated miR-542-5p suppressed OGT and OGA expression in SK0V3 cells. SKOV3 cells were cultured in 6 well plates and treated with vehicle, or KU60019 (10 μM) for 24 h. Then cells were collected and qPCR or flow cytometry analysis of miR-542-5p **(A)**, OGT **(B, D)**, and OGA **(C, E)** in SKOV3 cells. *P< 0.05; **P< 0.01; ***P<0.001; ****P < 0.0001. Data are representative of three independent experiments with similar results. Quantification of signal was shown in bar graphs and error bars represent mean ± SD.

To further investigate the relation between *miR-542-5p* and *OGT/OGA*, we reversed the downregulated expression of *miR-542-5p* under ATM inhibition by transfecting SKOV3 cells with corresponding miRNA mimics before KU60019 treatment. A sufficient increase in *miR-542-5p* levels was found in SKOV3 cells after treatment with the mimics, which could be restrained by adding KU60019 (**Figure 3A**). We also found a significant increase in the levels of *OGT* and *OGA* in SKOV3 cells after KU60019 treatment (an approximate 40% and 70% decrease difference could be detected in OGT and OGA expression, respectively), which was suppressed when *miR-542-5p* was upregulated (**Figures 3B,C**). In addition, we also detected similar changes at protein level, combination of KU60019 and *miR-542-5p* mimics reversed upregulation of OGT and OGA caused by KU60019 in SKOV3 cells (**Figures 3D,E**). These results further supported a regulatory role of *miR-542-5p* on *OGT* and *OGA* in SKOV3 cells under ATM inhibition.

### High Level of *miR-542-5p* Enhanced the Migration and Viability of SKOV3 Cells After KU60019 Treatment

**Figure 4:**
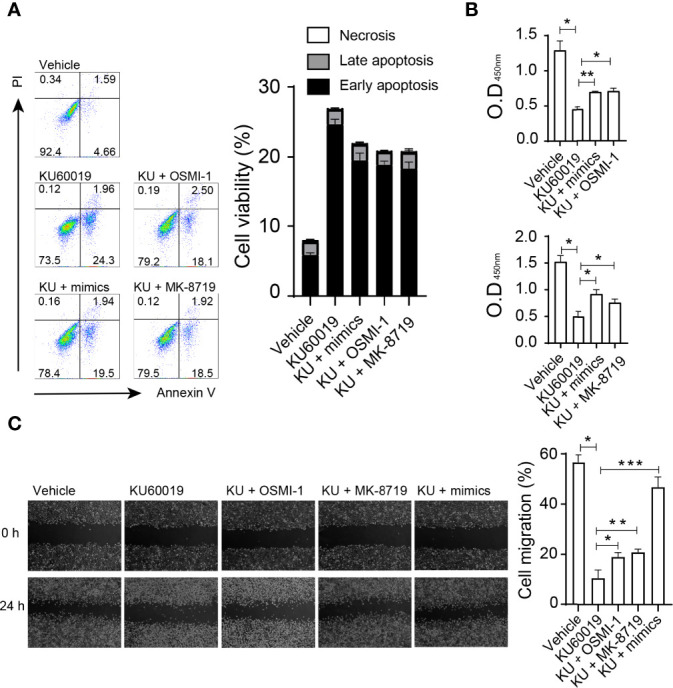
SKOV3 cells were cultured in 6 well plates and transfected with miR-542-5p mimic, or treated with KU60019 (10 μM), or MK-8719 (10 μM), or OSMI-1 (20 μM) for 24 h prior to wounding or apoptosis detection. Then cells were collected and stained with indicated reagents for apoptosis detection. SKOV3 cell apoptosis was examined by annexin V/PI staining **(A)**. **(B)** Detection of cell growth by CCK-8 assay. Images were taken of each well at indicating time-points. The width of the wound was measured three times per image **(C)**. *P< 0.05;**P< 0.01; ***P<0.001. Data are representative of three independent experiments with similar results. Quantification of signal was shown in bar graphs and error bars represent mean ± SD.

Next, we investigated the influence of the *miRNA-542-5p/OGT/OGA* axis on cellular behaviors. SKOV3 cells were cultured in 6 well plates and transfected with miR-542-5p mimic, or treated with KU60019 (10 μM), or OGT inhibitor OSMI-1 (20 μM), or OGA inhibitor MK-8719 (10 μM) for 24 hours. Administration of *miRNA-542-5p* mimics or OSMI-1, or MK-8719 effectively reversed the apoptosis of SKOV3 cells induced by KU60019 (**Figure 4A**). In addition, overexpression of *miRNA-542-5p*, or administration of OSMI-1, or MK-8719 significantly promoted the growth and migratory potential of SKOV3 cells (**Figures 4B,C**). These findings suggest that the *miRNA-542-5p/OGT/OGA* axis regulated apoptosis and migration of SKOV3 cells.

### Aberrant Expression of OGT Correlated With the Pathological Stage Ovarian Cancer Patients

**Figure 5:**
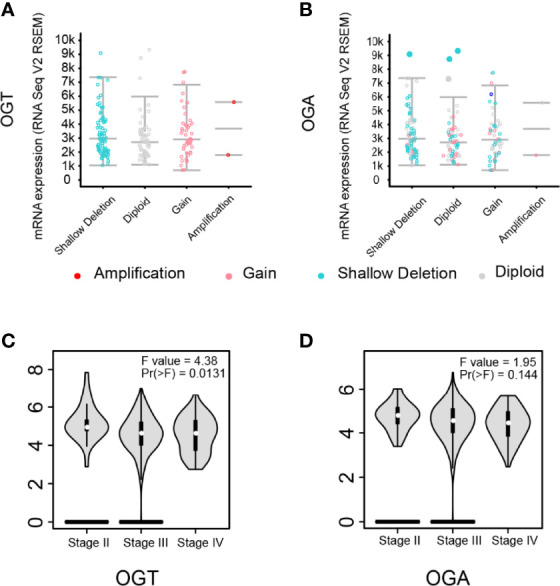
Aberrant expression of OGT was correlated with pathological stage in ovarian cancer. **(A-B)** Putative copy-number alterations of OGT/OGA. **(C-D)** The distribution of OGT mRNA expression correlated with tumor stage in ovarian cancer patients (p < 0.05).

To investigate the impact of OGT/OGA in ovarian cancer patients, we analyzed copy-number alterations of which from the cBioPortal for Cancer Genomics database and found that more up-regulation of OGT (**Figure 5A**) could be observed but not OGA(**Figure 5B**). More importantly, we found the aberrant expression of OGT was significantly correlated with the pathological stage in ovarian cancer patients (**Figure 5C**), while changes in OGA seems only have limited impact (**Figure 5D**). These results indicated that changes in OGT might correlate with ovarian cancer progression in patients.

### *OGT* and *OGA* Influence the Survival of Patients With Ovarian Cancer

**Figure 6:**
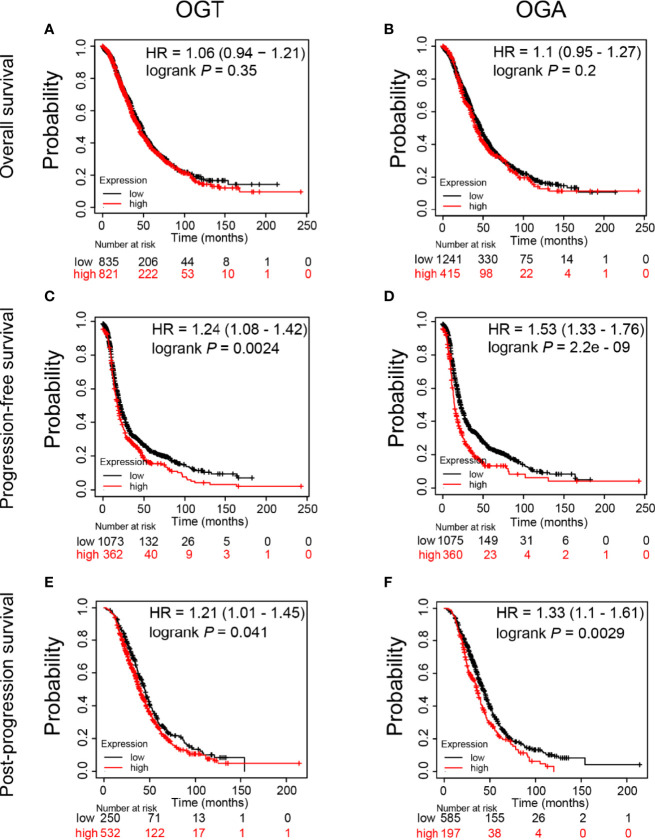
Prognostic value of mRNA expression of OGT and OGA in ovarian cancer patients. **(A-B)** Impact of OGT/OGA to OS of ovarian cancer patients. **(C-D)** Impact of OGT/OGA to PFS of ovarian cancer patients. **(E-F)** Impact of OGT/OGA to PPS of ovarian cancer patients.

Since significant alteration of *OGT* and *OGA* was observed in SKOV3 cells after KU60019 treatment, we next evaluated the prognostic value of these genes in ovarian cancer patients, including overall survival (OS), progression-free survival (PFS), and post-progression survival (PPS). We found that high level of *OGT or OGA* in ovarian cancer tissues has no significant correlation with OS (**Figures 6A,B**). In addition, increased *OGA* and *OGT* levels impaired PFS (**Figures 6C,D**), and shortened PPS in ovarian cancer patients (**Figures 6E,F**). Collectively, these results indicated that *OGT* and *OGA* could substantially influence the survival rate of ovarian cancer patients from several aspects.

## Discussion

ATM plays a key role in the DNA damage response pathway. Accumulating evidences from previous cancer studies highlights the repair function of ATM upon DNA double-strand breaks. Targeting ATM with an ATM inhibitor such as KU60019 is a promising strategy to inhibit tumor cell growth in antitumor therapy for several cancer types. However, the ATM inhibition’s influence in ovarian cancer treatment remained unclear. Here, we found that ATM inhibition could elevate the expression of *OGT* and *OGA* while significantly suppressing the level of *miR-542-5p* in ovarian cancer SKOV3 cells When *miR-542-5p* was overexpressed under ATM inhibition, *OGT* expression was effectively inhibited. These results indicated that *miR-542-5p* may target both *OGT* and *OGA* through three indispensable genes that regulate fat reserves and energy metabolism in SKOV3 cells.

Energy metabolism fluctuations strongly influence the progression, growth, and metastasis of cancer in different stages. Indeed, O-GlcNAcylation is a post-translational modification that transfers β-linked N-acetylglucosamine by integrating glucose, amino acids, and fatty acids by *OGT*, they are removed by *OGA*. Even slight cellular stresses can induce changes in O-GlcNAcylation signaling, thus altering the expression of tumor-associated proteins. Indeed, it is reported that inhibition of O-GlcNAclation of SNAP-23 could affect the exosome release by ovarian cancer cells, leads to changes in chemoresistance (27). *In vitro* experiments showed that migration and invasion ability could be effectively impaired in SKOV3 cells *via* suppressing OGT expression (28). Thiamet-G is an inhibitor for OGA, administration of Thiamet-G could activate p53 and up-regulate downstream proteins expression in A2780 and SKOV-3 cells, indicating a potential therapy target for ovarian cancer treatment (29). Aneta Rogalska and colleagues found that abnormal OGT expression could induce ovarian cancer cell apoptosis (30). As a result, *OGT* and *OGA* play a pivotal role in tumor progression and prognosis. In this study, we identified the aberrantly elevated expression of *OGT* and *OGA* as a consequence of KU60019 treatment in SKOV3 cells. These results are consistent with studies on colon cancer (31), intestinal tumorigenesis (32), and other types of tumorigeneses. Significant cell death was also found in SKOV3 cells after ATM inhibition, indicating a potential correlation between cell viability and energy metabolism-related genes. Our results showed that high expression of OGT/OGA significantly impaired the PFS and PPS in ovarian cancer patients. PFS could reflects tumor growth and can be evaluated before a survival benefit is proven, without being influenced by potentially confounding indicators or symptoms of subsequent therapy. In addition, the results of PFS appear earlier than those of survival, which may be an acceptable surrogate endpoint for clinical benefit in support of routine drug approval (33, 34). Post-progression survival could reflect survival post‐progression, which is critical in understanding treatment effects (35). Thus, our finding might help evaluate the efficacy of drugs in future clinical trials.

In addition to a disorder in metabolic enzyme genes, we identified *miR-542-5p* as a key regulatory gene in the presence of KU60019 in SKOV3 cells. Previous studies have shown that overexpressed *miR-542-5p* impaired the migration of glioblastoma U251 cells by downregulating the expression of *AEG-1*. *miR-542-5p* has also been shown to be essential in silicosis and breast cancer progression. However, the mechanism by which *miRNA* might influence SKOV3 cells in the presence of KU60019 is unknown. Our results strongly suggest that inhibition of *miR-542-5p* promotes SKOV3 cell apoptosis. Since upregulated expression of *miR-542-5p* in SKOV3 cell significantly improved cell migration and viability as compared to those of the control group, targeting *miR-542-5p* may represent a new promising approach to treat ovarian cancer. Elevated *miR-542-5p* expression was shown to substantially promote osteosarcoma growth. Similarly, we found that upregulation of *miR-542-5p* facilitated cell growth of SKOV3 cells. Based on these findings, we plan to further validate *miR-542-5p* as a therapeutic target for ovarian cancer, and study its underlying molecular mechanisms to provide theoretical support for future clinical application.

In conclusion, our results demonstrate that the expression of *OGT* and *OGA* is elevated in SKOV3 cells after KU60019 treatment, whereas *miR-542-5p* levels are decreased. Moreover, enhancing *miR-542-5p* expression reversed cell apoptosis in the presence of KU60019. We further show that *miR-542-5p* might target *OGT* and *OGA*, suggesting *miR-542-5p* as a novel target for ovarian cancer therapy. These findings provide new support for the conceptual premise that blocking ATM signaling might also influence the stability of the metabolic system in SKOV3 cells. Thus, our findings expand on the basic theory of the ATM inhibition as an anti-cancer strategy, and provide novel insight for therapeutic interventions in ovarian cancer. However, future studies are needed to explore the detailed molecular mechanism involved.

## Data Availability Statement

The raw data supporting the conclusions of this article will be made available by the authors, without undue reservation.

## Ethics Statement

The current study has been submitted to and approved by the ethics committee of the First Hospital of Jilin University.

## Author Contributions

NW performed the experiments. NW and MY analyzed the data and wrote the manuscript. NW collected samples. NW, ZM, and YF revised the manuscript. ZM and YF conceived the idea and supervised the project. All co-authors have seen and approved the manuscript.

## Funding

This work was supported by the Science and Technology Department of Jilin Province (20190201140JC to HY).

## Conflict of Interest

The authors declare that the research was conducted in the absence of any commercial or financial relationships that could be construed as a potential conflict of interest.

## Publisher’s Note

All claims expressed in this article are solely those of the authors and do not necessarily represent those of their affiliated organizations, or those of the publisher, the editors and the reviewers. Any product that may be evaluated in this article, or claim that may be made by its manufacturer, is not guaranteed or endorsed by the publisher.
